# Comparative Effectiveness of Psychotherapy vs Antidepressants for Depression in Heart Failure: A Randomized Clinical Trial

**DOI:** 10.1001/jamanetworkopen.2023.52094

**Published:** 2024-01-17

**Authors:** Waguih William IsHak, Michele A. Hamilton, Samuel Korouri, Marcio A. Diniz, James Mirocha, Rebecca Hedrick, Robert Chernoff, Jeanne T. Black, Harriet Aronow, Brigitte Vanle, Jonathan Dang, Gabriel Edwards, Tarneem Darwish, Gabrielle Messineo, Stacy Collier, Mia Pasini, Kaleab K. Tessema, John G. Harold, Michael K. Ong, Brennan Spiegel, Kenneth Wells, Itai Danovitch

**Affiliations:** 1 Department of Psychiatry and Behavioral Neurosciences, Cedars-Sinai Medical Center, Los Angeles, California; 2 David Geffen School of Medicine at UCLA, Los Angeles, California; 3 Smidt Heart Institute, Department of Cardiology, Cedars-Sinai Medical Center, Los Angeles, California; 4 Biostatistics Research Center, Cedars-Sinai Medical Center, Los Angeles, California; 5 Cedars-Sinai Health System, Los Angeles, California; 6 Department of Nursing Research, Cedars-Sinai Medical Center, Los Angeles, California; 7 Division of Health Services Research, Department of Medicine, Cedars-Sinai Health System, Los Angeles, California

## Abstract

**Importance:**

Heart failure (HF) affects more than 6 million adults in the US and more than 64 million adults worldwide, with 50% prevalence of depression. Patients and clinicians lack information on which interventions are more effective for depression in HF.

**Objective:**

To compare the effectiveness of behavioral activation psychotherapy (BA) vs antidepressant medication management (MEDS) on patient-centered outcomes inpatients with HF and depression.

**Design, Setting, and Participants:**

This pragmatic randomized comparative effectiveness trial was conducted from 2018 to 2022, including 1-year follow-up, at a not-for-profit academic health system serving more than 2 million people from diverse demographic, socioeconomic, cultural, and geographic backgrounds. Participant included inpatients and outpatients diagnosed with HF and depression, and data were analyzed as intention-to-treat. Data were analyzed from 2022 to 2023.

**Interventions:**

BA is an evidence-based manualized treatment for depression, promoting engagement in personalized pleasurable activities selected by patients. MEDS involves the use of an evidence-based collaborative care model with care managers providing coordination with patients, psychiatrists, and primary care physicians to only administer medications.

**Main Outcomes and Measures:**

The primary outcome was depressive symptom severity at 6 months, measured using the Patient Health Questionnaire 9-Item (PHQ-9). Secondary outcomes included physical and mental health-related quality of life (HRQOL), measured using the Short-Form 12-Item version 2 (SF-12); heart failure-specific HRQOL, measured using the Kansas City Cardiomyopathy Questionnaire; caregiver burden, measured with the Caregiver Burden Questionnaire for Heart Failure; emergency department visits; readmissions; days hospitalized; and mortality at 3, 6, and 12 months.

**Results:**

A total of 416 patients (mean [SD] age, 60.71 [15.61] years; 243 [58.41%] male) were enrolled, with 208 patients randomized to BA and 208 patients randomized to MEDS. At baseline, mean (SD) PHQ-9 scores were 14.54 (3.45) in the BA group and 14.31 (3.60) in the MEDS group; both BA and MEDS recipients experienced nearly 50% reduction in depressive symptoms at 3, 6, and 12 months (eg, mean [SD] score at 12 months: BA, 7.62 (5.73); *P* < .001; MEDS, 7.98 (6.06); *P* < .001; between-group *P* = .55). There was no statistically significant difference between BA and MEDS in the primary outcome of PHQ-9 at 6 months (mean [SD] score, 7.53 [5.74] vs 8.09 [6.06]; *P* = .88). BA recipients, compared with MEDS recipients, experienced small improvement in physical HRQOL at 6 months (mean [SD] SF-12 physical score: 38.82 [11.09] vs 37.12 [10.99]; *P* = .04), had fewer ED visits (3 months: 38% [95% CI, 14%-55%] reduction; *P* = .005; 6 months: 30% [95% CI, 14%-40%] reduction; *P* = .008; 12 months: 27% [95% CI, 15%-38%] reduction; *P* = .001), and spent fewer days hospitalized (3 months: 17% [95% CI, 8%-25%] reduction; *P* = .002; 6 months: 19% [95% CI, 13%-25%] reduction; *P* = .005; 12 months: 36% [95% CI, 32%-40%] reduction; *P* = .001).

**Conclusions and Relevance:**

In this comparative effectiveness trial of BA and MEDS in patients with HF experiencing depression, both treatments significantly reduced depressive symptoms by nearly 50% with no statistically significant differences between treatments. BA recipients experienced better physical HRQOL, fewer ED visits, and fewer days hospitalized. The study findings suggested that patients with HF could be given the choice between BA or MEDS to ameliorate depression.

**Trial Registration:**

ClinicalTrials.gov Identifier: NCT03688100.

## Key Points

**Question:**

Is behavioral activation psychotherapy (BA) or antidepressant medication management (MEDS) more effective in patients with heart failure experiencing depression?

**Findings:**

In this comparative effectiveness randomized clinical trial that included 416 adults, BA recipients as well as MEDS recipients experienced nearly 50% reduction of depressive symptoms at 3, 6, and 12 months, with no statistically significant differences between treatments. BA recipients experienced improvement in physical health-related quality of life, fewer ED visits, and fewer days hospitalized compared with MEDS recipients.

**Meaning:**

These findings suggest that patients with heart failure could be given the choice between BA or MEDS to ameliorate depression.

## Teaser

This comparative effectiveness randomized clinical trial compares the effectiveness of behavioral activation psychotherapy vs antidepressant medication management for patient-centered outcomes among patients with heart failure experiencing depression.

## Introduction

Heart failure (HF) affects more than 6 million adults in the US and more than 64 million adults worldwide.^1^ Depression is the leading cause of disability worldwide; yet, it is largely undiagnosed and untreated in the HF population.^2^ Approximately 50% of people with HF experience depressive symptoms, due to an interplay of biological and psychosocial mechanisms.^2,3,4^ Patients with HF and depression have lower cardiac function,^5^ more emergency department (ED) visits and hospital admissions,^6^ higher caregiver burden,^7^ and poorer health-related quality of life (HRQOL)^8^ compared with patients with HF without depression. Depressive symptom severity is a greater risk factor for poor HRQOL than HF symptom severity,^8^ associated with increased risks for functional decline or death at 6 months,^9^ and an independent factor associated with all-cause mortality.^10^ However, only 50% of patients who are diagnosed with depression and HF receive depression treatment.^3^

The American Heart Association recommends screening for depression in patients with cardiovascular disease (CVD).^11^ However, the value of screening is predicated on the availability of evidence-based treatments, such as antidepressant medications and psychotherapy, which patients often have particular difficulty accessing.^12^ Psychotherapy interventions include cognitive behavioral therapy (CBT), which has shown efficacy for depression in patients with HF,^13^ and behavioral activation psychotherapy (BA),^14^ which is as efficacious as CBT^15^ but more feasible in patients with HF.^16^ Pharmacotherapy using antidepressant medications has also established efficacy for depression in patients with HF.^10^ However, clinicians and patients lack evidence on which intervention to use for depression in HF. Therefore, we sought to compare the effectiveness of psychotherapy vs pharmacotherapy for patient-centered outcomes in a randomized trial reflecting the challenges facing clinicians, patients, and caregivers.

In this context, we conducted a randomized trial to compare the efficacy of BA^17^ vs antidepressant medication management (MEDS)^10^ in patients with HF and depression. Outcomes of interest included depressive symptom severity, physical and mental HRQOL, HF-specific HRQOL, caregiver burden, morbidity (as measured by ED visits, hospital readmissions, and days hospitalized), and mortality at 3, 6, and 12 months.^18^ We hypothesized that BA would be superior to MEDS on both primary and secondary outcomes, as suggested by previous research^17^ and given its lack of contribution to medication burden.

## Methods

This comparative effectiveness randomized clinical trial was approved by the Cedars-Sinai institutional review board. The trial protocol^18^ and statistical analysis plan are available in Supplement 1. All participants provided written informed consent. This study is reported following the Consolidated Standards of Reporting Trials (CONSORT) reporting guideline.

### Trial Design and Oversight

We conducted a randomized comparative effectiveness trial of 2 evidence-based treatments for depression in inpatients and outpatients with HF: BA vs MEDS. Our trial protocol^18^ was developed in collaboration with an advisory group of clinicians (cardiologists, primary care physicians, psychiatrists, and psychotherapists), researchers, patients, caregivers, and patient advocates. Based on evaluation using the PRECIS-2 tool,^19^ the study was performed in a pragmatic fashion, as detailed in eAppendix 1 in Supplement 2. The study was monitored by a data safety monitoring board, as detailed in eAppendix 2 in Supplement 2).

### Patients and Participating Centers

This study was conducted from 2018 to 2022 within Cedars-Sinai Health System, a not-for-profit academic health system in California serving more than 2 million people with diverse demographic, socioeconomic, cultural, and geographic (urban, suburban, and rural) characteristics. Study participants were recruited from inpatients admitted to a hospital with HF and outpatients presenting to clinics for HF follow-up, were given adequate information and time to think about the study, and signed a written informed consent. The study sample included patients with HF (New York Heart Association class II, III, or IV) and a life expectancy of more than 6 months (confirmed by the treating physician) who were experiencing depressive symptoms (Patient Health Questionnaire-9 [PHQ-9] score ≥10) with a confirmed *Diagnostic and Statistical Manual of Mental Disorders* (Fifth Edition) (*DSM-5*) depressive disorder diagnosis by the Mini International Neuropsychiatric Interview for *DSM-5*. Patients were excluded if they were exhibiting imminent danger to self or others (by clinical assessment); were experiencing cognitive impairments (Montreal Cognitive Assessment score <23); had bipolar, psychotic, or substance-induced disorders (by Mini International Neuropsychiatric Interview); or were in active treatment for depression (antidepressants and/or psychotherapy). We enrolled patient-identified family or friend caregivers, after they signed a written informed consent, to measure caregiver burden.

### Interventions and Trial Procedures

BA is an evidence-based manualized treatment for depression promoting engagement in personalized pleasurable activities selected by patients.^17^ BA has established efficacy in depression in more than 25 randomized clinical trials,^14^ with comparable effectiveness with CBT.^15^ The emphasis in BA is on improving patient mood, sense of control, and activity level by increasing engagement in pleasurable and rewarding tasks without delving into complex cognitive domains explored in CBT, which makes it more suitable for patients with HF.^16^ BA has lower implementation barriers due to high uptake by patients and feasibility of administration by various health care professionals with less expensive and lengthy training.^15,17,20^

MEDS involved the administration of antidepressants^10^ using the evidence-based collaborative care model (CCM).^21,22^ To preserve treatment comparison, care managers did not provide psychotherapy in this study as in classic CCM. Care managers provided the CCM key components of population-based care, measurement-based care, team-based care, and psychiatric consultation to facilitate evidence-based MEDS. We incorporated the American Psychological Association antidepressant guidelines and World Health Organization antidepressant equivalence dosing guidelines to address the issue of using a variety of antidepressants to avoid limiting the trial to a single antidepressant, which would not only contradict clinical practice but also would limit the research to 1 medicine that might not suit every patient due to side effects, drug-drug interactions, and efficacy.

Participants meeting study criteria were randomly assigned in a 1:1 ratio to BA or MEDS using the REDCap randomization module (Vanderbilt University), and assigned to either a licensed social worker as a BA therapist following BA guidelines^20^ to provide BA psychotherapy, as detailed in eAppendix 3 in Supplement 2, or a registered nurse as an MEDS care manager following CCM^21^ guidelines to provide MEDS only, as detailed in eAppendix 4 in Supplement 2. After receiving 2 hours of training on their roles, treatment techniques, and study procedures with subsequent 1.5-hour weekly meetings, BA therapists and MEDS care managers facilitated a 50-minute introductory session followed by 12 weekly sessions (BA: 50-minute sessions or MEDS: 15-minute sessions), then monthly for 3 months, followed by contact as needed for an additional 6 months.

We delivered BA and MEDS interventions using video or telephone because the disease burden of patients with HF tends to impair their ability to attend in-person sessions. Evidence suggests that telehealth reduces barriers to psychiatric treatments by saving patient and caregiver time and improving adherence, in addition to mood and HRQOL improvement, without compromising efficacy.^23^ Similar to other research and clinical centers, we experienced major disruption due to COVID-19, affecting patients regardless of whether they were randomized to BA or MEDS. However, our study continued recruitment from inpatient (in person) and from outpatient (using electronic consent forms). In accordance with the original study design, follow up surveys were completed using telehealth. Anecdotally, we actually experienced an easier time reaching patients at home due to COVID-19 lockdowns, for the purposes of recruitment, electronic consent, treatment delivery, and outcome collection.

Intervention fidelity was ensured by study coinvestigators by conducting weekly meetings with BA therapists and MEDS care managers and reviewing patients’ involvement. Moreover, with patient consent, the study psychologist listened in on at least 1 BA session provided by each therapist to check on fidelity, with random checks as needed. MEDS used a similar procedure with fidelity checklists.

Adherence to assigned interventions was measured by tracking the number and length of sessions and completion of assigned intervention components. Study procedures are further detailed in eAppendix 5 in Supplement 2.

### Outcomes

The primary outcome was depressive symptom severity at 6 months, measured by the PHQ-9 (range, 0-27; higher score indicates more severe depression).^24^ Secondary outcomes were collected at 3, 6, and 12 months and included 3 measures of patient HRQOL, 1 measure of caregiver burden, 3 measures of morbidity, and mortality. Physical and mental HRQOL were measured by the 12-Item Short-Form Medical Outcomes Study (SF-12, version 2; range 0-100; higher score indicates better HRQOL).^25^ HF-specific HRQOL was assessed using the 23-item patient-reported Kansas City Cardiomyopathy Questionnaire (KCCQ; range 0-100; higher score indicates better HRQOL).^26^ Caregiver burden was assessed using the caregiver-reported 26-item Caregiver Burden Questionnaire for Heart Failure (CBQ-HF; range 0-130; higher score indicates more severe burden).^27^ Morbidity was measured by ED visits, hospital readmissions (admissions for outpatients and readmissions for inpatients and outpatients), and days hospitalized by self or caregiver report. Mortality data were collected from medical records and family member or caregiver reports.

Covariates included age, sex, race (self-reported), ethnicity (self-reported), marital status, employment, educational level, insurance type, recruitment site (inpatient or outpatient), ejection fraction (EF; categorized as preserved or reduced), NYHA class, medical history, and medications (eAppendix 6 in Supplement 1). Race was reported as African-American or Black, Asian, White, or other (ie, participant did not identify with any of the race categories), and ethnicity was reported as Hispanic or Latinx or not Hispanic or Latinx. Race and ethnicity were assessed because Race and ethnicity were assessed to examine whether racial and ethnic factors play a role in treatment response differences.

### Statistical Analysis

Summary statistics were presented as counts with percentages for categorical variables and means with SDs and medians with IQRs for numerical variables. The primary analysis was intention-to-treat. Analyses of PHQ-9 at 6 months (primary outcome) and other questionnaires were performed using multivariable linear mixed models^28^ considering baseline response, time, treatment, and interaction between time and treatment as covariates with random effects, describing repeated measures at 3, 6, and 12 months after treatment.^29^ Missing data were imputed using multivariate imputation by chained equations with 20 imputed data sets.^30^ ED visits, readmissions, and days hospitalized, as functions of treatment, used zero-inflated Poisson (ZIP) regression models.^31^ Survival was analyzed using multivariable Cox proportional hazards models. Kaplan-Meier survival plots were assessed for 3, 6, and 12 months. Tests of hypotheses were 2-sided, using α = .05 significance level. Statistical analyses were implemented using SAS software version 9.4 (SAS Institute), and R software version 4.1.2 (R Project for Statistical Computing). Data were analyzed from 2022 to 2023.

## Results

**Figure 1.**
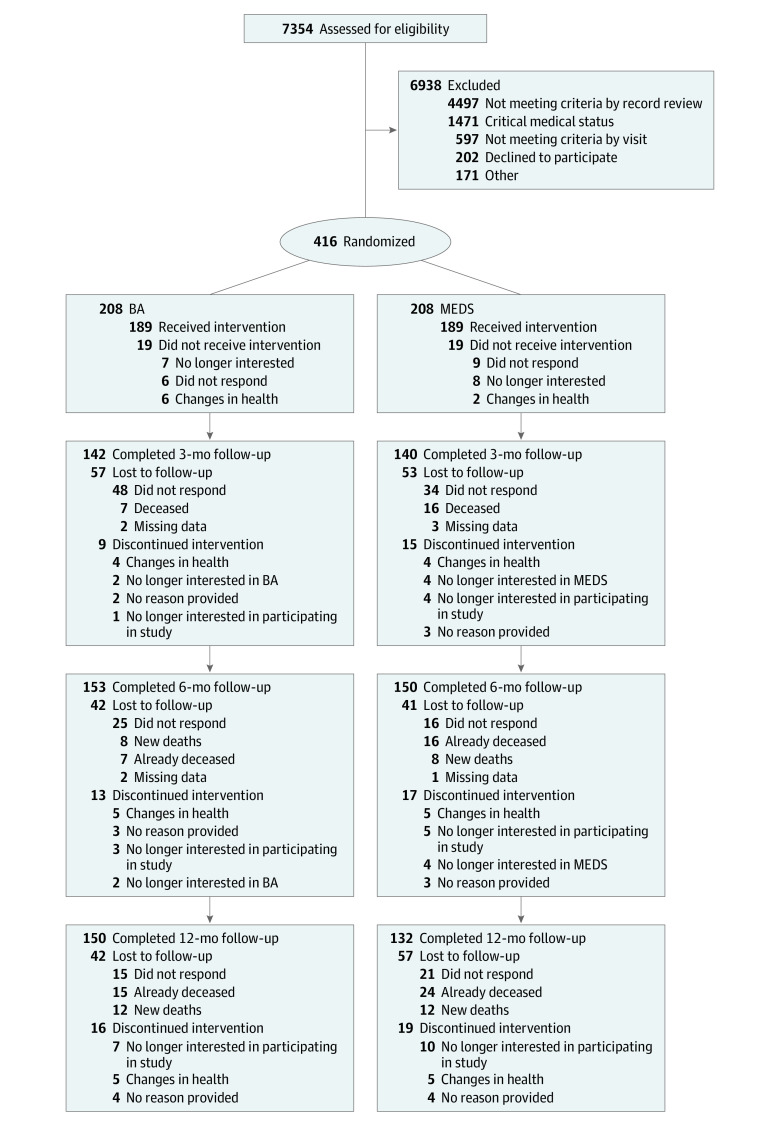
Participant Enrollment Flowchart BA indicates behavioral activation psychotherapy; MEDS antidepressant medication management.

A total of 416 patients (mean [SD] age, 60.71 [15.61] years; 243 [58.41%] male) were enrolled, with 208 patients randomized to BA and 208 patients randomized to MEDS. Figure 1 details the flow of patients through the study starting with the number assessed for eligibility, patients excluded and why, and participants lost to follow-up after 3, 6, and 12 months of treatment. Baseline characteristics for the 416 patients randomized are presented in Table 1. The total sample included 122 African-American or Black participants (29.33%), 21 Asian participants (5.05%), and 233 White participants (56.01%); 60 participants (14.42%) were Hispanic or Latinx and 344 participants (82.69%) were not Hispanic or Latinx. There were no significant differences in demographic characteristics between participants randomized to BA vs MEDS (Table 1). Among clinical characteristics, there was a higher prevalence of atrial fibrillation and use of anticoagulants and digoxin in the BA group and a higher prevalence of chronic liver disease and history of antidepressant use in the MEDS group (Table 1), which were all accounted for in statistical analyses. There was no significant difference in PHQ-9 score between the BA group (mean [SD] score, 14.54 [3.45]) and the MEDS group (mean [SD] score, 14.31 [3.60]) at baseline. Among the 78 caregivers enrolled, we found no baseline CBQ-HF score differences between BA and MEDS.

**Table 1.** Demographic and Clinical Characteristics at Baseline

Variable	Participants, No. (%)		
	Overall (N = 416)	BA (n = 208)	MEDS (n = 208)
Age, y			
Mean (SD)	60.71 (15.61)	60.84 (15.69)	60.59 (15.57)
Median (range)	62 (18.00-94)	64 (20-94)	61 (18-89)
Sex			
Female	173 (41.59)	88 (42.31)	85 (40.87)
Male	243 (58.41)	120 (57.69)	123 (59.13)
Other	0	0	0
Recruitment location			
Inpatient	206 (49.52)	99 (47.60)	107 (51.44)
Outpatient	210 (50.48)	109 (52.40)	101 (48.56)
Race			
African-American or Black	122 (29.33)	62 (29.81)	60 (28.85)
Asian	21 (5.05)	7 (3.37)	14 (6.73)
White	233 (56.01)	121 (58.17)	112 (53.85)
Other^a^	40 (9.62)	18 (8.65)	22 (10.58)
Ethnicity			
Hispanic or Latinx	60 (14.42)	28 (13.46)	32 (15.39)
Not Hispanic or Latinx	344 (82.69)	173 (83.17)	171 (82.21)
Not reported	12 (2.89)	7 (3.37)	5 (2.40)
Marital status			
Married	169 (40.62)	86 (41.35)	83 (39.90)
Single	140 (33.65)	66 (31.73)	74 (35.58)
Divorced or separated	77 (18.51)	40 (19.23)	37 (17.79)
Widowed	30 (7.21)	16 (7.69)	14 (6.73)
Education			
College degree	95 (22.84)	45 (21.63)	50 (24.04)
High school	103 (24.76)	56 (26.92)	47 (22.60)
Some college	153 (36.78)	73 (35.10)	80 (38.46)
Graduate degree	65 (15.62)	34 (16.35)	31 (14.90)
Employment status			
Disabled	142 (34.13)	68 (32.69)	74 (35.58)
Unemployed	47 (11.30)	23 (11.06)	24 (11.54)
Employed	91 (21.88)	43 (20.67)	48 (23.08)
Retired	136 (32.69)	74 (35.58)	62 (29.81)
BMI			
Mean (SD)	28.94 (8.27)	29.07 (8.80)	28.81 (7.73)
Median (IQR) [range]	27.40 (22.98-33.20) [15.10-85.00]	27.35 (23.00-33.62) [15.10-85.00]	27.50 (22.90-32.92) [16.40- 58.00]
PHQ-9^b^			
Mean (SD)	14.42 (3.52)	14.54 (3.45)	14.31 (3.60)
Median (range)	14 (100-24.)	14 (10-23)	14 (10-24)
SF-12 physical component score^c^			
Mean (SD)	35.72 (8.52)	35.89 (9.26)	35.56 (7.74)
Median (range)	34.91 (16.41-67.39)	34.38 (19.33-67.39)	35.19 (16.41-56.15)
SF-12 mental component score^c^			
Mean (SD)	37.04 (9.19)	36.87 (9.16)	37.22 (9.23)
Median (range)	36.54 (9.68-64.00)	36.54 (14.83-63.69)	36.52 (9.68-64.00)
KCCQ overall summary score^c^			
Mean (SD)	40.21 (21.17)	39.88 (22.08)	40.54 (20.28)
Median (range)	37.24 (0.00-96.35)	37.50 (0.00-96.35)	36.85 (2.34-91.67)
KCCQ clinical summary score^c^			
Mean (SD)	43.05 (23.32)	42.43 (24.14)	43.68 (22.51)
Median (range)	40.63 (0.00-100.00)	39.58 (0.00-100.00)	40.63 (3.13-100.00)
Medical conditions			
Hypertension	285 (68.51)	143 (68.75)	142 (68.27)
Diabetes	157 (37.74)	80 (38.46)	77 (37.02)
Obstructive sleep apnea	157 (37.74)	86 (41.35)	71 (34.13)
Atrial fibrillation or atrial flutter	246 (59.13)	137 (65.87)	109 (52.40)
Myocardial infarction	148 (35.58)	80 (38.46)	68 (32.69)
Chronic obstructive pulmonary disease	62 (14.90)	35 (16.83)	27 (12.98)
Percutaneous coronary intervention	138 (33.17)	75 (36.06)	63 (30.29)
Coronary artery bypass graft	56 (13.46)	33 (15.87)	23 (11.06)
Stroke or TIA	85 (20.43)	48 (23.08)	37 (17.79)
Implantable cardiac defibrillator	139 (33.41)	74 (35.58)	65 (31.25)
Pacemaker	102 (24.52)	60 (28.85)	42 (20.19)
Valvular disease	117 (28.12)	56 (26.92)	61 (29.33)
Peripheral vascular disease	39 (9.38)	22 (10.58)	17 (8.17)
Hypothyroidism	79 (18.99)	43 (20.67)	36 (17.31)
Chronic kidney disease	135 (32.45)	60 (28.85)	75 (36.06)
Chronic liver disease	64 (15.38)	24 (11.54)	40 (19.23)
Rheumatoid arthritis	78 (18.75)	44 (21.15)	34 (16.35)
Collagen vascular disease	18 (4.33)	8 (3.85)	10 (4.81)
Obesity	148 (35.58)	81 (38.94)	67 (32.21)
Anemia	171 (41.11)	89 (42.79)	82 (39.42)
Charlson Comorbidity Index			
Mean (SD)	5.49 (2.23)	5.54 (2.30)	5.44 (2.17)
Median (IQR) [range]	5.50 (4.00-7.00) [1.00-12.00]	6.00 (4.00-7.00) [1.00-11.00]	5.00 (4.00-7.00) [1.00-12.00]

Abbreviations: BA, behavioral activation psychotherapy; BMI, body mass index (calculated as weight in kilograms divided by height in meters squared); KCCQ, Kansas City Cardiomyopathy Questionnaire; MEDS, antidepressant medication management; PHQ-9, Patient Health Questionnaire–9-Item; SF-12, Short Form–12 Item; TIA, transient ischemic attack.

^a^ Participant did not identify with any of the race categories.

^b^ Range 0-27; higher score indicates more severe depression.

^c^ Range 0-100; higher score indicates better quality of life.

### Outcomes

#### Depressive Symptom Severity at 6 Months

**Figure 2.**
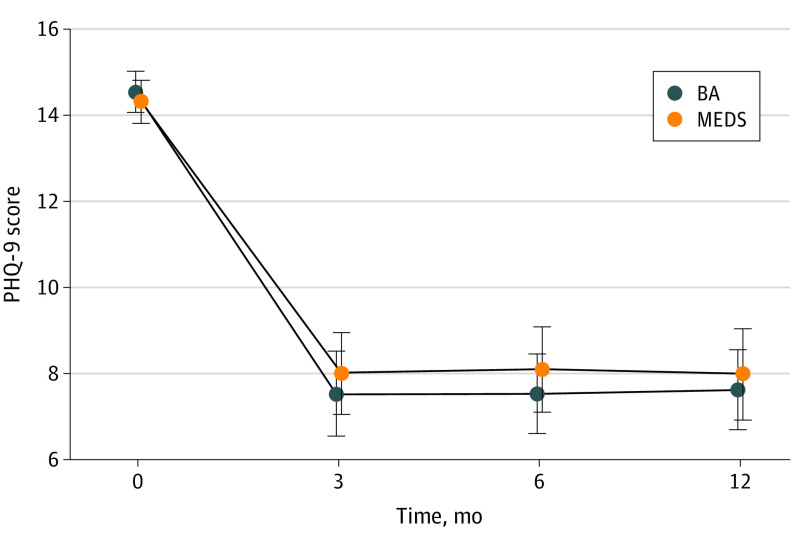
Depressive Symptom Severity at Baseline, 3, 6, and 12 Months The primary outcome was 9-item Patient Health Questionnaire (PHQ-9) score at 6 months. Differences between behavioral activation psychotherapy (BA) and antidepressant medication management (MEDS) groups were not statistically significant.

Depressive symptom severity was reduced at 6 months by nearly 50% for both BA (mean [SD] PHQ-9 score, 7.53 [5.74]; *P *vs baseline < .001) and MEDS (mean [SD] PHQ-9 score, 8.09 [6.06]; *P *vs baseline < .001) participants with no significant difference between BA and MEDS (mean PHQ-9 difference without imputation ,−0.10 [95% CI, −0.33 to 1.13]; *P* = .88; mean PHQ-9 difference with imputation, −0.07 [95% CI, −1.28 to 1.13]; *P* = .70) (Figure 2).

#### Secondary Outcomes

**Figure 3.**
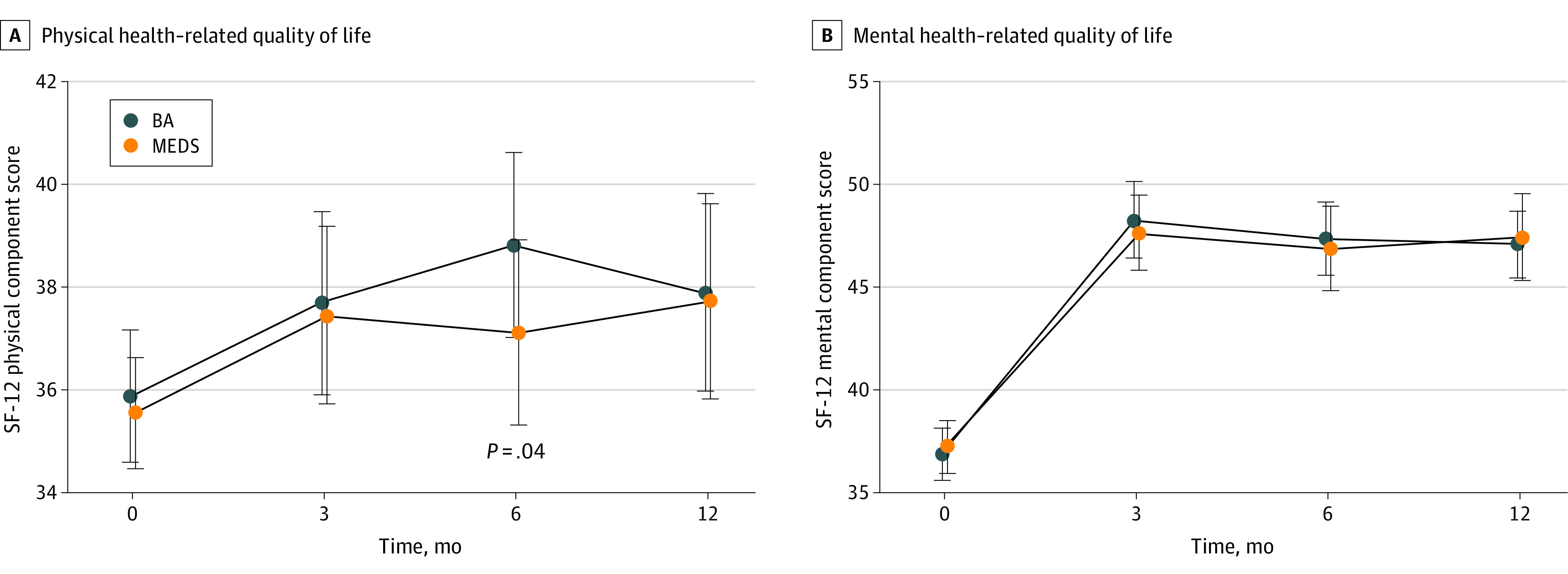
Health-Related Quality of Life, Physical and Mental Components The difference between behavioral activation psychotherapy (BA) and antidepressant medication management (MEDS) groups was statistically significant only on the Short-Form–12-Item (SF-12) physical component at 6 months (mean difference, 2.13 [95% CI, 0.06-4.20]; *P* = .04).

Compared with baseline, statistically significant improvements were detected in both BA and MEDS groups on the secondary outcomes of physical HRQOL, mental HRQOL, and HF-specific QOL at 6 months. Compared with MEDS recipients, BA recipients experienced improvement in physical HRQOL, as measured by the SF-12, at 6 months (multivariable mean difference without imputation, 2.13 [95% CI, 0.06-4.20]; *P* = .04). This difference is higher than the minimal clinically important difference of 2 established for the SF-12 by Ware et al^32^ and used in heart disease research.^33^ There were no statistically significant differences between BA and MEDS groups in physical HRQOL at 3 or 12 months (Figure 3).

There were no statistically significant differences between BA and MEDS groups in mental HRQOL, HF-specific HRQOL, or caregiver burden at 3, 6, or 12 months (Table 2). Using per-protocol analyses, we compared patients who were mostly adherent to each intervention using data on BA and MEDS session completion as well as data on completing the BA tasks or using prescribed MEDS. At the 6-month time point only, BA patients, compared with MEDS patients, had significantly better physical HRQOL, as well as significantly better HF-specific HRQOL. Adherence data collection is described in eAppendix 7 in Supplement 2.

**Table 2.** Primary and Secondary Outcome Scores at Baseline and 3, 6, and 12 Months

Measure	Baseline, mean (SD)		3 mo			6 mo			12 mo		
	BA	MEDS	Mean (SD)		*P* value^a^	Mean (SD)		*P* value^a^	Mean (SD)		*P* value^a^
			BA	MEDS		BA	MEDS		BA	MEDS	
PHQ-9^b^	14.54 (3.45)	14.31 (3.60)	7.53 (5.90)	8.00 (5.62)	.69	7.53 (5.74)	8.09 (6.06)	.88	7.62 (5.73)	7.98 (6.06)	.55
SF-12^c^											
Physical	35.89 (9.26)	35.56 (7.74)	37.69 (10.60)	37.46 (10.17)	.27	38.82 (11.09)	37.12 (10.99)	.04	37.89 (11.74)	37.72 (10.81)	.22
Mental	36.87 (9.16)	37.22 (9.23)	37.69 (10.60)	37.46 (10.17)	.89	47.33 (10.97)	46.85 (12.55)	.98	47.04 (9.91)	47.41 (12.07)	.68
KCCQ^c^											
Overall	39.88 (22.08)	40.54 (20.28)	62.03 (25.69)	60.57 (23.03)	.29	64.35 (26.89)	61.43 (24.48)	.20	63.24 (25.88)	65.48 (24.83)	.76
Clinical	42.43 (24.14)	43.68 (22.51)	64.01 (26.76)	64.16 (24.88)	.39	66.15 (27.17)	63.39 (25.92)	.11	64.00 (27.19)	67.02 (24.43)	.75
CBQ-HF^d^	65.14 (20.96)	68.06 (20.04)	57.70 (21.62)	61.52 (19.72)	.85	55.27 (18.13)	57.38 (17.96)	.55	60.39 (24.98)	63.19 (23.63)	.38

Abbreviations: BA, behavioral activation psychotherapy; CBQ-HF, Caregiver Burden Questionnaire for Heart Failure; KCCQ, Kansas City Cardiomyopathy Questionnaire; MEDS, antidepressant medication management; PHQ-9, Patient Health Questionnaire–9-Item; SF-12, Short Form–12 Item.

^a^ The significance of differences between BA and MEDS shown are from multivariable regression without imputation. In the univariate models, all *P* values were greater than .05.

^b^ Range 0-27; higher score indicates more severe depression.

^c^ Range 0-100; higher score indicates better quality of life.

^d^ Range 0-130; higher score indicates more severe burden.

Compared with patients who received MEDS, patients who received BA were significantly less likely to have ED visits at 3, 6, and 12 months. Using ZIP models, the means ratio for ED visits for BA to MEDS was 0.62 (95% CI, 0.45-0.86) at 3 months (*P* = .005), 0.70 (95% CI, 0.60-0.86) at 6 months (*P* = .008), and 0.73 (95% CI, 0.62-0.85) at 12 months (*P* = .001). There were no significant differences in number of hospital readmissions for BA and MEDS groups using ZIP models at 3, 6, and 12 months. BA patients spent fewer days hospitalized at 3, 6, and 12 months with BA vs MEDS means ratios of 0.83 (95% CI, 0.75-0.92) at 3 months (*P* = .002), 0.81 (95% CI, 0.75-0.87) at 6 months (*P* = .005), and 0.64 (95% CI, 0.60-0.68) at 12 months (*P* = .001).

There were no statistically significant differences in mortality between BA and MEDS groups at 3, 6, or 12 months based on Kaplan-Meier survival plots. In multivariable Cox regression models, significant risk factors associated with mortality were inpatient vs outpatient site of enrollment and patients with NYHA class III and IV compared with NYHA class II.

### Subgroup Analyses

We predefined 3 sets of subgroups, based on the literature, for which analyses were performed: (1) baseline depressive symptom severity, defined by PHQ score (moderate, 10-14; moderately severe, 15-19; severe, 20-27)^34^; (2) HF type, defined by EF (reduced EF, ≤40%; preserved EF, >40%)^35^; and (3) HF symptom severity, defined by NYHA classes (Class II, III, and IV).^36^ No statistically significant differences were detected between BA and MEDS groups in any subgroups.

### Safety

There were no significant adverse events during the study. Deaths were deemed unrelated to the study interventions by the data safety monitoring board.

## Discussion

This comparative effectiveness randomized clinical trial of BA vs MEDS found that among patients with HF and depression, there was reduced depressive symptom severity by nearly 50% in both interventions at 6 months, with no significant differences between BA and MEDS groups at 3, 6, or 12 months. There were no significant differences in secondary outcomes of HRQOL, caregiver burden, morbidity, or mortality between BA and MEDS groups at 3, 6, or 12 months. However, patients receiving BA, compared with those receiving MEDS, had slightly higher physical HRQOL improvement at 6 months and were less likely to visit the ED with fewer days hospitalized at 3, 6, and 12 months, which were all statistically significant. There was no significant difference in hospital readmissions. Patients in BA were encouraged to be physically active to a degree that patients in the MEDS condition may not have been. This may have contributed to their tendency to have fewer ED visits and hospital days, in contrast to hospital readmissions that are more related to poor general health, which is the most reliable risk factor associated with rehospitalization in patients with HF and depression.^37^

Our findings are consistent with studies comparing BA with antidepressants in the general population.^22^ The finding that patients in MEDS had a 50% reduction in depressive symptom severity at 3 months and sustained the reduction at 6 and 12 months is consistent with reports from the IMPACT study, which found that 45% of patients receiving collaborative care had a significant reduction in depressive symptoms over 12 months.^21^ Our findings ran counter to the results of a meta-analysis suggesting that BA is superior to medication treatment for depression^17^; however, studies in the meta-analysis by Ekers et al had shorter follow-ups than our study. Concerning depression in patients with HF, several studies have assessed the efficacy of various psychosocial interventions. For example, a study by Rollman et al^36^ used 12-month telephone-delivered collaborative care compared with usual care for patients with HF and depression and showed improvements in mood and mental HRQOL but no differences in hospital readmissions or mortality.^36^ Bekelman et al^38^ compared a collaborative care intervention with usual care, finding improved depression but not HF-specific HRQOL at 6 months and no differences in readmissions or mortality at 12 months.^38^ Additionally, multiple studies have shown that CBT^12,13,39,40,41,42^ and exercise therapy,^42^ are superior to placebo and/or usual care for treating depression in patients with HF. However, to our knowledge, no studies directly compared MEDS and BA in heart disease.

### Applicability and Generalizability

For patients with HF and depression, the decision of whether to pursue psychotherapy or medication represents a common dilemma. BA requires more engagement and effort but has the benefit of no pharmacological adverse effects. MEDS requires less time and effort but presents risks of adverse effects and drug interactions. In the absence of comparative effectiveness evidence, decision-making can be subject to influence by misconceptions and stigma.^43^ Our findings of comparable primary effects between BA and MEDS suggest both options are effective and that personal preferences, patient values, and availability of services could inform decisions, thus offering patients, caregivers, and clinicians choices between treatments.^44^ The improvement in physical HRQOL, lower likelihood of ED visits, and fewer hospitalization days observed in the BA group compared with the MEDS group suggest secondary advantages for BA, especially that patients with HF and depression may be reluctant to add more medications to their already large pill burden. In fact, many patients report that they would prefer nonpharmacological treatment over more medications,^45^ especially psychotherapy.^46^ Yet, it is important to acknowledge that it is more difficult in everyday practice to obtain evidence-based psychotherapies as opposed to antidepressants.^47^ Finally, the pragmatic nature of all aspects of our study and intervention delivery (based on PRECIS-2 analysis),^19^ and the diverse populations in this study suggest findings are generalizable to a wide range of patients with HF in inpatient and outpatient settings.

### Strengths

The study was conducted in a clinical environment with its inpatient and outpatient components and the involvement of cardiologists, primary care physicians, and collaboration with mental health professionals. This carried significant strengths. The study’s strengths include its pragmatic design, sample size, diverse population, recruitment from both inpatient and outpatient settings, and relevance to practice through the engagement of patient and practitioner advisory groups. It is important to emphasize that although research shows that women as well as African American or Black adults are underrepresented in clinical trials despite their high risk of CVD,^48,49^ this trial enrolled higher ratios compared with literature-reported CVD trials, with nearly 42% women (vs 38% in CVD trials),^48^ and 30% African American or Black patients (vs <29% in 90% of CVD trials).^49^ BA has the advantage of low staff training demand, contributing to improved feasibility. The study’s 1-year follow-up added more support to the findings. Conducting interventions by telehealth was important to ease the treatment burden of HF, especially during COVID-19.^50^ This also offers a viable and sustainable treatment delivery mode as telehealth continues in the postpandemic era.^51^

### Limitations

There were several study limitations. There was no control group, such as no treatment or waiting list, so it is not possible to draw conclusions about the natural course of depressive symptoms in HF. However, improvements were not only detected at 3 months (after 12 weekly sessions), and 6 months (after 3 monthly additional sessions), but sustained at 12 months (despite substantially diminished contact with intervention teams after 6 months). We were unable to collect data for ED visits, readmissions, and hospital stays outside of California. It would have been helpful to assess treatment preference at enrollment to inform its association with outcomes and adherence. Per-protocol analyses comparing BA with MEDS based on adherence to the interventions are expected to be finalized and shared in future publications. Future research might need to explore which patients benefited from which treatment.

## Conclusions

In this comparative effectiveness randomized clinical trial of BA and MEDS for depression in patients with HF, both treatments significantly reduced depressive symptoms but were not significantly different in primary depression severity outcome, although BA had advantages for some secondary outcomes. To our knowledge, this was the first comparative effectiveness trial of BA vs MEDS for depression in patients with HF. Our findings demonstrate that both interventions are comparably effective in reducing depression for patients with HF, giving patients, caregivers, and health care practitioners the choice between BA and MEDS, thus improving patient-centered depression care in HF.

## Notes

Supplement 1.
Trial Protocol and Statistical Analysis Plan

Supplement 2. **eAppendix 1.** PRECIS-2 Tool for Pragmatic Trials **eAppendix 2.** DSMB Monitoring and Advisory Board Engagement **eAppendix 3.** Guidelines for BA **eAppendix 4.** Guidelines for MEDS **eAppendix 5.** Study Procedures **eAppendix 6.** Covariates **eAppendix 7.** Adherence and Treatment Dose
eReferences.
**eTable 1.** PRECIS-2 Tool Results **eTable 2.** BA Session Guidelines **eTable 3.** Antidepressant Medication Guidelines **eTable 4.** Guidelines for MEDS Care Managers and Supervising Psychiatrists **eTable 5.** Covariate Definitions

Supplement 3.
Data Sharing Statement
