# Supplementary material for: Comparative Effectiveness of Psychotherapy vs Antidepressants for Depression in Heart Failure: A Randomized Clinical Trial
Source: JAMA Netw Open. 2024 Jan 17;7(1):e2352094. doi: 10.1001/jamanetworkopen.2023.52094 (PMC10794938; doi:10.1001/jamanetworkopen.2023.52094)
Supplement: Supplement 2. — eAppendix 1. PRECIS-2 Tool for Pragmatic Trials eAppendix 2. DSMB Monitoring and Advisory Board Engagement eAppendix 3. Guidelines for BA eAppendix 4. Guidelines for MEDS eAppendix 5. Study Procedures eAppendix 6. Covariates eAppendix 7. Adherence and Treatment Dose eReferences. eTable 1. PRECIS-2 Tool Results eTable 2. BA Session Guidelines eTable 3. Antidepressant Medication Guidelines eTable 4. Guidelines for MEDS Care Managers and Supervising Psychiatrists eTable 5. Covariate Definitions [file jamanetwopen-e2352094-s002.pdf]

Supplemental Online Content

IsHak WW, Hamilton MA, Korouri S, et al. Comparative effectiveness of psychotherapy vs antidepressants for depression in heart failure: a randomized clinical trial. *JAMA Netw Open*. 2024;7(1):e2352094. doi:10.1001/jamanetworkopen.2023.52094

eAppendix 1. PRECIS-2 Tool for Pragmatic Trials
eAppendix 2. DSMB Monitoring and Advisory Board Engagement
eAppendix 3. Guidelines for BA
eAppendix 4. Guidelines for MEDS
eAppendix 5. Study Procedures
eAppendix 6. Covariates
eAppendix 7. Adherence and Treatment Dose
eReferences.

This supplemental material has been provided by the authors to give readers additional information about their work.

© 2024 IsHak WW et al. *JAMA Network Open.*

eAppendix 1 – PRECIS-2 Tool for pragmatic trials.

Based on evaluation using the PRECIS-2 tool,[1] the study was performed in a pragmatic fashion as detailed in eTable 1 below.

eTable 1 –PRECIS-2 analysis to evaluate pragmatic design using nine domains (https://www.precis-2.org)
Scoring: 5-point Likert scale (1=Very explanatory, 2=Rather explanatory, 3=Equally pragmatic and explanatory, 4=Rather pragmatic, 5=Very pragmatic)

DOMAIN	QUESTION	SCORE
**Eligibility**	To what extent are the participants in the trial similar to those who would receive this intervention if it was part of usual care?	5 - participants in this trial were recruited from admitted inpatients and outpatients with heart failure who screened positive for depression and were identical to those who would receive pharmacotherapy (MEDS) or psychotherapy (BA) in usual care.
**Recruitment**	How much extra effort is made to recruit participants over and above what would be used in the usual care setting to engage with patients?	5 - we recruited participants from inpatients and outpatients as well as reached out to primary care physicians and cardiologists to recruit patients from regularly scheduled patients at diverse sites. We did not advertise or make extra efforts over and above what would be used in a usual care setting to engage patients who were not receiving usual care.
**Setting**	How different are the settings of the trial from the usual care setting?	4 - as the settings of the trial were not very different from the usual care setting where pharmacotherapy (MEDS) or psychotherapy (BA) would be administered with one exception, which is the use of telehealth, which has now become one of the standards of delivering these treatments during the COVID era.
**Organization**	How different are the resources, provider expertise, and the organization of care delivery in the intervention arm of the trial from those available in usual care?	4 - the resources, provider expertise, and the organization of care delivery of pharmacotherapy (MEDS) or psychotherapy (BA) were not very different from those available in usual care setting with the following exceptions: a) the availability of training of pharmacotherapy (MEDS) care managers by supporting psychiatrists is not available in usual care specialty cardiology care, but is available primary care settings; b) the availability of psychotherapy training (BA) therapists by supporting psychologists is not available in usual care specialty cardiology care, but is available primary care settings.
**Flexibility (delivery)**	How different is the flexibility in how the intervention is delivered and the flexibility anticipated in usual care?	5 - the flexibility in how pharmacotherapy (MEDS) or psychotherapy (BA) was delivered was not different from usual care. Personalized BA and MEDS were delivered by therapists and care managers respectively, still within guidelines such as the BA original manual and the MEDS guidelines for dosing. Fidelity assessments were conducted to ensure that participants received the standard of delivery of each intervention as detailed in the protocol.
**Flexibility (adherence)**	How different is the flexibility in how participants are monitored and encouraged to adhere to the intervention	5 - the flexibility in how participants were monitored and encouraged to adhere to pharmacotherapy (MEDS) or psychotherapy (BA) was not different from usual care. Examples of such flexibility in our trial included: not using any pre-trial screening to test adherence, and not terminating

© 2024 IsHak WW et al. *JAMA Network Open.*

	from the flexibility anticipated in usual care?	any participant who was non-adherent to the frequency, length of BA or MEDS sessions, or with BA tasks or MEDS medication intake. We continued to encourage adherence as it is done in usual care while tracking and monitoring adherence for data analysis purposes.
**Follow-up**	How different is the intensity of measurement and follow-up of participants in the trial from the typical follow-up in usual care?	4 - we did not collect extensive data during visits, and included measures that are collected in usual care follow-up visits such as the PHQ-9, the KCCQ which is widely used in patients with heart disease, and information on ED visits and hospital readmissions, as well as adherence to pharmacotherapy (MEDS) or psychotherapy (BA). Patients were contacted if they missed their appointments, as is done in usual care.
**Primary outcome**	To what extent is the trial's primary outcome directly relevant to participants?	5 - we designed and executed the study with a main focus on depressive symptom severity as measured by the patient-reported PHQ-9, which is the outcome that is most and directly relevant to patients.
**Primary analysis**	To what extent are all data included in the analysis of the primary outcome?	5 - all data were included in the analysis using the Intent-to-Treat principle which preserved the notion of "As randomized, so analyzed" and we used data imputation as needed.

© 2024 IsHak WW et al. *JAMA Network Open.*

eAppendix 2 – Advisory Board engagement and DSMB monitoring.

DSMB monitoring

The Personalized Treatments for Depressive Symptoms in Heart Failure study utilized a Data and Safety Monitoring Board (DSMB) to ensure study participant safety and data quality. The DSMB consisted of four physicians who were independent from the research team and who had experience in community intervention studies. The DSMB met every three months, and on an as-needed basis if necessary, to discuss participant safety concerns, review procedures and decisions regarding subject protection, protocol deviations, serious adverse events and adverse events, review study progress to ensure recruitment and other research goals have been met, to monitor and provide advice on scientific and ethical issues related to the study, and to review and oversee data confidentiality, data quality, data management, and data analysis. In the event of emerging data produced by the study or found through the scientific literature, the DSMB had the authority to recommend study discontinuation, modification, or termination. As part of the Cedars-Sinai Medical Center IRB's yearly continuing review process, the DSMB made recommendations regarding the continuation or conclusion of the study. All serious adverse events were reported to the DSMB and evaluated by its members for any concerns related to study involvement.

Advisory Board engagement

The Personalized Treatments for Depressive Symptoms in Heart Failure study utilized a stakeholder-academic advisory board to reinforce the pragmatic intent of the study design. The advisory board included two physicians with expertise in community participatory research, a consultant with expertise in psychotherapy, patient representatives, caregivers of patients with heart failure, and other professionals from diverse fields connected to mental health and cardiology. In the early phases of the study, the advisory group provided input to inform the study design, interventions, and outcomes, as well as strategies for implementation and adaptation. Thereafter, the advisory group met every month to discuss the study progress, recruitment, and provide further input for any challenges faced during the study. Near the end of the study, the advisory group continued to meet every quarter to discuss outcomes collected, data analysis, and plan for study dissemination and implementation efforts. It was crucial for us to significantly involve this advisory board in the conduct of our study, to ensure the scientific and stakeholder relevance of our research.

© 2024 IsHak WW et al. *JAMA Network Open.*

eAppendix 3 – Guidelines for BA.

The Behavioral Activation (BA) intervention utilizes an adaptation of the Brief Behavioral Activation Treatment for Depression, Revised Treatment Manual (BATD-R).[2] A study psychologist trains therapists in the BATD-R manual and holds weekly supervision meetings to discuss therapists' cases and ensure accurate delivery of the BA treatment. BA Therapists record individual session completion in a BA Patient Tracking Log, and record patients' engagement with the BA intervention in a BA Survey. See eTable 2 below for session-by-session guidelines for the BA intervention.

eTable 2 – BA session guidelines

BA session number	Session Guidelines
Session 1	Patients receive psychoeducation about depression and reasons for how BA could help counteract depression. Patients are assigned a Daily Monitoring Form on which they are tasked to describe how they spend their time each day and rate the enjoyment and importance of each of their activities on a 0-10 rating scale.
Session 2	Therapists review patients' Daily Monitoring Forms and discuss and troubleshoot any challenges patients face in completing the forms. Therapists then introduce and discuss the Life Areas, Values and Activities Form to patients. Patients are asked to identify and explore five areas of their lives: (1) relationships, (2) education/career/volunteering, (3) recreation and interests, (4) mind/body/spirituality, and (5) daily responsibilities. Therapists help patients articulate their values in each of the five life areas. From their identified values, patients are asked to identify specific activities that represent expressions of their values in each of these areas. Patients are asked to continue tracking their activities for the coming week on the Daily Monitoring Forms.
Sessions 3 and 4	Therapists continue helping patients define and refine their values and the specific activities linked to their values which they would find enjoyable and important. Therapists help patients develop a list of 15 activities from which to choose and schedule for the coming weeks. Patients are encouraged to select activities in all five areas that are observable and measurable, and to break activities down into their smallest parts. Using the Activities Selection and Ranking Form, patients rank their proposed 15 activities from the least to the most difficult. Patients then use the Daily Monitoring Form to plan the activities they are to engage in during the coming weeks.
Sessions 5 and 6	Using the Contracts Form, therapists encourage patients to enlist other people in their social support system to help them engage in valued activities. Patients continue to report what activities they engaged in during the previous week, what challenges they faced pursuing their activities, and activities planned for the coming week.
Sessions 7 through 9	Therapists continue to review the previous week's activities with patients, their ratings of enjoyment and importance for each activity, and activities planned for the coming week. In addition, therapists review each of the concepts and principles from the earlier sessions, including the concept of the five life domains, their values in each area, and the activities linked to their values. Therapists review the idea of selecting and ranking different activities, using them to plan and schedule activities for the coming week, and soliciting the help of other people to engage in valued and enjoyable activities.

© 2024 IsHak WW et al. *JAMA Network Open.*

Sessions 10 through 12	Patients continue reporting on the previous week's scheduled activities, their enjoyment and importance, any challenges they experienced, and planned activities for the coming week. As session 12 is the last weekly session, therapists explain that their next contact with the patient is going to be three consecutive monthly sessions.<br>Patients are encouraged to continue scheduling and engaging in enjoyable and meaningful activities on their own.
Monthly follow-up sessions	Patients report their activities and any challenges they encounter. At the third and last follow-up session, patients are encouraged to continue engaging in activities on their own. Patients are also told that they are welcome to contact their therapist on an as-needed basis if they run into obstacles and feel that they need additional coaching to continue engaging in valued and enjoyable activities.

© 2024 IsHak WW et al. *JAMA Network Open.*

eAppendix 4 – Guidelines for MEDS.

The Antidepressant Medication Management (MEDS) intervention utilizes the collaborative care model (CCM)[3] to start the patient on appropriate antidepressant medication. At the introductory session, a care manager collects patient and caregiver preferences regarding medication options. During 12 weekly telehealth visits, 3 monthly visits, and any as-needed visits thereafter, a care manager tracks patients' progress with their antidepressant medication and assesses whether medication changes need to be made. If so, the care manager coordinates communication between the patient's assigned study psychiatrist and their treating physician to ensure appropriate medication changes are made. To preserve treatment comparison, care managers did not provide psychotherapy in this study as in classic CCM. MEDS care managers provided the other CCM key components of population-based care, measurement-based care, team-based care, and psychiatric consultation to facilitate evidence-based antidepressant medication management. A study psychiatrist conducts weekly supervision meetings to discuss care managers' cases and ensure accurate delivery of the MEDS intervention. MEDS care managers record individual session completion and patients' adherence to their antidepressant medication in a MEDS tracking log. eTable 3 below details the guidelines used in the MEDS intervention to select an appropriate antidepressant medication.

eTable 3 – Antidepressant medication guidelines

Order	Medication class	Side effects	Interacting Agents common to HF	Clinical Effect & Monitoring	Recommended Medication and minimum therapeutic dose
1st line	SSRIs (sertraline, paroxetine, fluoxetine, escitalopram)	Sexual dysfunction, GI: decreased appetite, nausea, diarrhea, constipation, dry mouth. CNS: insomnia, sedation, agitation, tremors, headache, dizziness.	NSAIDs, oral anticoagulants, antiplatelets<br><br>Diuretics<br><br>Clonidine	Increased risk for bleeding<br><br>Hyponatremia<br><br>Hypothermia, sedation	Sertraline (50mg/day)
2	Dopaminergic and norepinephrine agents (bupropion)	Dry mouth, GI: constipation, nausea CNS: insomnia, dizziness, headache, agitation, anxiety. Weight loss, anorexia, myalgia, tremor, sweating, rash, hypertension	Clonidine<br><br>Warfarin	Hypothermia, sedation<br><br>Increased international normalized ratio	Bupropion SR (150 mg twice/day) recommended in HF patients with high BMI.<br><br>OR hypotension
2	Noradrenergic and serotonergic agents (mirtazapine)	Flulike symptoms, changes in urinary symptoms, weight gain, GI: decreased appetite, nausea, diarrhea, constipation, dry mouth, CNS: sedation, confusion. Hypotension*	Clonidine<br><br>Warfarin	Hypothermia, sedation<br><br>Increased international normalized ratio	Mirtazapine (15 mg at bedtime) recommended in HF patients with low BMI.<br><br>OR Hypertension<br>OR if patient is unresponsive to SSRIs or DNRIs
3	SNRI (venlafaxine, duloxetine)	Sexual dysfunction, GI: decreased appetite, nausea, diarrhea, constipation, dry	NSAIDs, oral anticoagulants, antiplatelets	Increased risk for bleeding	Use if patient was unresponsive to SSRI, DNRI or mirtazapine.

© 2024 IsHak WW et al. *JAMA Network Open.*

		mouth. CNS: insomnia, sedation, headache. hyponatremia, hypertension			Venlafaxine (225mg/day)<br><br>Duloxetine (60 mg/day)
X	TCAs	Orthostatic hypotension, tachycardia, QT prolongation, weight gain			Not recommended for HF patients
X	MAOIs	Orthostatic hypotension, hypertension bradycardia, weight gain			Not recommended for HF patients

eTable 4: Guidelines for MEDS Care Managers and Supervising Psychiatrists

1	1. Start medication according to guidelines and conversation between the primary care provider, patient and caregiver (12 weeks).
2	2. If side effects occur:<br>Consider drug-drug interactions:<br>a. serotonin syndrome: antidepressants, opioids, stimulants, 5-HT1 agonists, herbs, mood stabilizers, antipsychotics, antiemetics (ondansetron, metoclopramide), antibiotics (linezolid)<br>b. QTc Prolongation: SSRIs, antipsychotics, opioids, macrolides, fluoroquinolones, antiarrhythmics
3	3. Stop and start a new medication if side effects persist (10-14 days) after starting use, except sexual dysfunction
4	4. Refer to PI/Co-I in the event of:<br>a. refractory mood symptoms<br>b. development of psychotic and or manic symptoms<br>c. suicidal ideation<br>*following will be reported to the Data & Safety Monitoring Board (DSMB)<br>* PI/Co-I will be available after hours and patients will be given a 24-hour number to an on-call Psychiatrist

Abbreviations: HF: Heart Failure, SSRIs: Selective Serotonin Reuptake Inhibitors, GI: Gastrointestinal, CNS: Central Nervous System, NSAIDs: Non-Steroidal Anti-Inflammatory Drugs, DNRIs: Norepinephrine and Dopamine Reuptake Inhibitors, TCAs: Tricyclic Antidepressants, MAOIs: Monoamine Oxidase Inhibitors

© 2024 IsHak WW et al. *JAMA Network Open.*

eAppendix 5 – Study procedures

Potential study participants were identified via referrals from clinicians, through a Cedars-Sinai Medical Center IRB-approved artificial intelligence software, and a Cedars-Sinai Medical Center registry of heart failure patients. Patients were approached at the bedside when hospitalized with the approval of their treating physician; or were called over the phone if they were discharged or referred as an outpatient. All patients were asked if they had a caregiver who would like to participate in the study. At baseline, patients were screened to see if they met all study inclusion criteria and did not meet any study exclusion criteria, and patients' ejection fraction and New York Heart Association class were determined via chart review and/or by conferring with the patient's treating physician. All patients provided informed consent, and demographics and medical history were collected. Patients were randomized to via the Research Electronic Data Capture system (REDCap)3 to the Behavioral Activation (BA) psychotherapy intervention, or to the Antidepressant Medication Management (MEDS) intervention. If a patient was randomized to BA, they were assigned to work with a therapist, who was a licensed social worker supervised by a psychologist who was a study investigator. If a patient was randomized to MEDS, they were assigned to work with a care manager, who was a registered nurse supervised by a psychiatrist who was a study investigator. Both the BA and MEDS interventions consisted of an in-person or remote 50-minute introductory session followed by 12 weekly remote treatment sessions (BA=50minute-sessions) or MEDS=15 minute-sessions), 3 monthly remote treatment sessions, and then contact on an as-needed basis for the final 6 months. Study outcomes were collected remotely by phone at baseline, 3-month, 6-month, and 12-month time points, by a trained outcome collector registered nurse of licensed social worker who was not involved in the patient's treatment. The outcomes collected included Depressive Symptom Severity using the 9-item PHQ, Physical and Mental HRQoL as measured by the 12-item Short Form Medical Outcomes Study (SF-12.v2), Heart Failure-specific HRQoL using the 23-item patient-reported Kansas City Cardiomyopathy Questionnaire (KCCQ), Caregiver Burden using the caregiver-reported 26-item Caregiver Burden Questionnaire for Heart Failure (CBQ-HF), Morbidity as measured by ED visits, hospital readmissions, and days hospitalized, and Mortality data were collected from medical records in addition to self/caregiver reports. Hospital readmissions were defined as admissions for outpatients and readmissions for inpatients and outpatients, during each outcome collection interval. If the patient stated that they had a caregiver who would like to participate in the study, the caregiver burden questionnaire was administered as part of the outcome collection. All outcomes were stored in REDCap. Some participants stated they no longer wished to participate in their assigned intervention but agreed to receive outcome calls, and as such patient-reported outcomes were collected for these participants. Some participants stated that they no longer wished to participate in either their assigned intervention or the outcome calls, and as such the study team ceased to contact these participants. Study dropouts, deaths, and patients lost to follow-up were recorded by members of the study team. Completeness of data was ensured through re-engagement efforts by the study team, as well as through obtaining data through chart review, if available and appropriate to do so. Study patients were compensated with $100 for the completion of baseline measures, and their caregivers were compensated with $25 for the completion of baseline measures. Study patients were compensated with $50 for each 3, 6, and 12-month measure that they completed, and their caregivers were compensated with $10 for each 3, 6, and 12-month measure that they completed.

© 2024 IsHak WW et al. *JAMA Network Open.*

eAppendix 6 – Covariates

eTable 5 – Covariate definitions

Covariate	Values	Definition
Age	Numerical value (years)	Participant's age
Sex	Female<br>Male<br>Other	Participant's sex
Race	American Indian/Alaskan Native<br>Asian<br>Black/African American<br>Caucasian/White<br>Native Hawaiian/Other Pacific Islander<br>Not Reported<br>Unknown<br>Other	Participant's race
Ethnicity	Hispanic or Latino<br>Not Hispanic or Latino<br>Not Reported<br>Unknown	Participant's ethnicity
Marital Status	Single<br>Married<br>Divorced<br>Widowed<br>Separated	Participant's marital status
Employment	Employed Full time<br>Employed Part time<br>Unemployed, looking for work<br>Unemployed, NOT looking for work<br>Retired due to age<br>Disabled<br>Other (e.g. student)	Participant's employment status
Educational Level	No High School<br>High School, no diploma<br>High School, diploma or GED<br>Some College, no degree<br>Associates Degree (Occupational, technical, vocational, academic program)<br>Bachelor's degree (e.g. BA, BS)<br>Master's degree (e.g. MA, MS, MBA, Med)<br>Professional/Doctoral Degree (e.g. MD, DDS, JD, PhD, EdD)	Level of education attained by the participant
Insurance		
Insurance Payor Name	Text value	Participant's insurance payor name
Insurance Plan Name	Text value	Participant's insurance plan name
Insurance Category	Self-pay (uninsured)<br>Medi-Cal<br>Medicare<br>Commercial<br>Military	Participant's insurance category
Recruitment Site	Inpatient<br>Outpatient	Location in which participant was recruited for the study
Ejection Fraction	Reduced ejection (≤40%)<br>Preserved ejection (>40%)	Participant's ejection fraction category

© 2024 IsHak WW et al. *JAMA Network Open.*

NYHA Class	Class I<br>Class II<br>Class III<br>Class IV	Participant's heart failure severity as represented by their New York Heart Association class
Medical History		
BMI	Numerical value	Participant's Body Mass Index calculated as (weight in kilograms)/(height in meters)
Hypertension	Yes<br>No	Whether or not the participant has hypertension
Diabetes	Yes<br>No	Whether or not the participant has diabetes
Obstructive Sleep Apnea	Yes<br>No	Whether or not the participant has obstructive sleep apnea
AFIB or atrial flutter	Yes<br>No	Whether or not the participant has AFIB or atrial flutter
Myocardial Infarction	Yes<br>No	Whether or not the participant has had a myocardial infarction
Chronic obstructive pulmonary disease	Yes<br>No	Whether or not the participant has Chronic obstructive pulmonary disease
Percutaneous coronary intervention	Yes<br>No	Whether or not the participant has had a percutaneous coronary intervention
Coronary artery bypass graft	Yes<br>No	Whether or not the participant has had a coronary artery bypass graft
Stroke or TIA	Yes<br>No	Whether or not the participant has had a stroke or TIA
Implantable cardiac defibrillator	Yes<br>No	Whether or not the participant has an implantable cardiac defibrillator
Pacemaker	Yes<br>No	Whether or not the participant has a pacemaker
Valvular Disease	Yes<br>No	Whether or not the participant has a valvular disease
Peripheral Vascular Disease	Yes<br>No	Whether or not the participant has a peripheral vascular disease
Hypothyroidism	Yes<br>No	Whether or not the participant has hypothyroidism
Chronic Renal (Kidney) Disease	Yes<br>No	Whether or not the participant has chronic renal disease
Chronic Liver Disease	Yes<br>No	Whether or not the participant has a chronic liver disease
Rheumatoid arthritis	Yes<br>No	Whether or not the participant has rheumatoid arthritis
Collagen Vascular Disease	Yes<br>No	Whether or not the participant has collagen vascular disease
Obesity	Yes<br>No	Whether or not the participant has obesity
Anemia	Yes<br>No	Whether or not the participant has anemia
Smoking Status	Past<br>Current<br>Unknown<br>Never Smoked	Participant's usage of tobacco products
History of Depression	Yes<br>No	Whether or not the participant has ever been diagnosed with depression

© 2024 IsHak WW et al. *JAMA Network Open.*

Treatment with antidepressant medication	Yes<br>No	Whether or not the participant has ever been treated with antidepressant medication
Treatment with Psychotherapy	Yes<br>No	Whether or not the participant has ever been treated with Psychotherapy
History of Anxiety Disorder	Yes<br>No	Whether or not the participant has ever been diagnosed with anxiety disorder
History of PTSD	Yes<br>No	Whether or not the participant has ever been diagnosed with PTSD
Medications		
Angiotensin Receptor Blocker	Numerical value (integer)	Quantity of Angiotensin Receptor Blocker medications the participant is on
Loop Diuretic	Numerical value (integer)	Quantity of Loop Diuretic medications the participant is on
Opiate	Numerical value (integer)	Quantity of opiate medications the participant is on
Angiotensin-Converting Enzyme Inhibitor	Numerical value (integer)	Quantity of Angiotensin-Converting Enzyme Inhibitor medications the participant is on
B-Blocker	Numerical value (integer)	Quantity of B-Blocker medications the participant is on
Aldosterone Antagonist	Numerical value (integer)	Quantity of Aldosterone Antagonist medications the participant is on
Calcium Channel Blocker	Numerical value (integer)	Quantity of Calcium Channel Blocker medications the participant is on
Digoxin	Numerical value (integer)	Quantity of Digoxin medications the participant is on
Steroid	Numerical value (integer)	Quantity of Steroid medications the participant is on
Cannabinoid	Numerical value (integer)	Quantity of cannabinoid medications the participant is on
Antidepressant	Numerical value (integer)	Quantity of Antidepressant medications the participant was previously on
Anticoagulant	Numerical value (integer)	Quantity of Anticoagulant medications the participant is on
Ivabradine	Numerical value (integer)	Quantity of Ivabradine medication the participant is on
Non-Loop Diuretic	Numerical value (integer)	Quantity of Non-Loop Diuretic medications the participant is on
Statin	Numerical value (integer)	Quantity of Statin medications the participant is on
Antiaggregant	Numerical value (integer)	Quantity of Antiaggregant medications the participant is on
Antiarrhythmic	Numerical value (integer)	Quantity of Antiarrhythmic medications the participant is on
Angiotensin Receptor Neprilysin Inhibitor (ARNI)	Numerical value (integer)	Quantity of ARNI medications the participant is on
Hydralazine and Nitrate	Numerical value (integer)	Quantity of Hydralazine and Nitrate medications the participant is on
Sodium-Glucose-Co-Transporter-2 (SGLT2) Inhibitor	Numerical value (integer)	Quantity of SGLT2 inhibitor medications the participant is on

© 2024 IsHak WW et al. *JAMA Network Open.*

eAppendix 7 –Adherence and Treatment Dose

Data on treatment adherence was also collected at 3, 6 and 12 months using two methods. The first method was adherence as measured by the percentage of sessions completed, and the second method was adherence as measured by the percentage of intervention content received.

For the BA arm, the percentage of intervention content received was measured by assessing the number of sessions during which the patient reported adhering to the tasks expected of them for BA. These expected tasks included completing the daily monitoring forms, logging activities, rating the importance/enjoyability of activities, and engaging in the activities that they designated as enjoyable and/or important to them.

For the MEDS arm, the percentage of intervention content received was measured by assessing the number of sessions during which the patient reported taking the minimum therapeutic dose of their prescribed antidepressant medication. The therapeutic dose was defined in eTable 3 according to the APA guidelines,[5] and the WHO antidepressant equivalence dosing guidelines.[6] Adherence for the MEDS arm was based on the collected Brief Medication Questionnaire,[7] in each MEDS session by the care managers. We collected MEDS adherence data from care manager chart notes into the electronic medical record; and we collected BA adherence data from the BA Tracking Log and BA Survey, which were two Excel spreadsheets where BA therapists recorded the details of their patients' sessions.

© 2024 IsHak WW et al. *JAMA Network Open.*

eReferences

1. Loudon K, Treweek S, Sullivan F, Donnan P, Thorpe KE, Zwarenstein M. The PRECIS-2 tool: designing trials that are fit for purpose. BMJ. 2015 May 8;350:h2147.

2. Lejuez CW, Hopko DR, Acierno R, Daughters SB, Pagoto SL. Ten year revision of the brief behavioral activation treatment for depression: revised treatment manual. *Behavior modification*. 2011; 35(2):111–161. https://doi.org/10.1177/0145445510390929 PMID: 21324944

3. Unutzer J, Katon W, Callahan CM, Williams JW, Hunkeler E, Harpole L et al. Collaborative care management of late-life depression in the primary care setting: a randomized controlled trial. *JAMA*. 2002;288(22):2836–2845. https://doi.org/10.1001/jama.288.22.2836 PMID: 12472325

4. Harris PA, Taylor R, Thielke R, Payne J, Gonzalez N, Conde JG. Research electronic data capture (REDCap) – a metadata-driven methodology and workflow process for providing translational research informatics support. *Journal of biomedical informatics*. 2009; 42(2):377-381. https://doi.org/10.1016/j.jbi.2008.08.010 PMID: 18929686

5. American Psychiatric Association. *Practice guideline for the treatment of patients with Major Depressive Disorder*. 2010. https://psychiatryonline.org/pb/assets/raw/sitewide/practice_guidelines/guidelines/mdd-1410197717630.pdf

6. WHO Collaborating Centre for Drug Statistics. *Anatomical Therapeutic Chemical (ATC)/Defined Daily Dose (DDD) classification system. Antidepressants*. 2020. https://www.whocc.no/atc_ddd_index/?code=N06A

7. Svarstad BL, Chewning BA, Sleath BL, Claesson C. The Brief Medication Questionnaire: a tool for screening patient adherence and barriers to adherence. *Patient Educ Couns.* 1999 Jun;37(2):113-24. https://10.1016/s0738-3991(98)00107-4 PMID: 14528539.

© 2024 IsHak WW et al. *JAMA Network Open.*
