# Supplementary material for: Comparative Effectiveness of Psychotherapy vs Antidepressants for Depression in Heart Failure: A Randomized Clinical Trial
Source: JAMA Netw Open. 2024 Jan 17;7(1):e2352094. doi: 10.1001/jamanetworkopen.2023.52094 (PMC10794938; doi:10.1001/jamanetworkopen.2023.52094)
Supplement: Supplement 3. — Data Sharing Statement [file jamanetwopen-e2352094-s003.pdf]

# Data Sharing Statement

IsHak. Comparative Effectiveness of Psychotherapy vs Antidepressants for Depression in Heart Failure. *JAMA Netw Open*. Published January 17, 2024.
doi:10.1001/jamanetworkopen.2023.52094

**Data**
**Data available:** Yes
**Data types:** Deidentified participant data
**How to access data:** Waguih.IsHak@cshs.org
**When available:** beginning date: 01-01-2025, end date: 06-30-2025

**Supporting Documents**
**Document types:** None

**Additional Information**
**Who can access the data:** Researchers whose proposed use of the data has been approved.
**Types of analyses:** For a specified purpose.
**Mechanisms of data availability:** After approval of a proposal.
**Any additional restrictions:** None.
